# Effective recruitment of participants to a phase I study using the internet and publicity releases through charities and patient organisations: analysis of the adaptive study of IL-2 dose on regulatory T cells in type 1 diabetes (DILT1D)

**DOI:** 10.1186/s13063-015-0583-7

**Published:** 2015-03-11

**Authors:** James Heywood, Marina Evangelou, Donna Goymer, Jane Kennet, Katerina Anselmiova, Catherine Guy, Criona O’Brien, Sarah Nutland, Judy Brown, Neil M Walker, John A Todd, Frank Waldron-Lynch

**Affiliations:** JDRF/Wellcome Trust Diabetes and Inflammation Laboratory, Department of Medical Genetics, NIHR Cambridge Biomedical Research Centre, Cambridge Institute for Medical Research, University of Cambridge, Wellcome Trust/MRC Building, Cambridge Biomedical Campus, Cambridge, CB2 0XY UK; The Cambridge BioResource, University of Cambridge and Cambridge University Hospitals NHS Foundation Trust, Cambridge Biomedical Campus, Cambridge, CB2 0QQ UK

**Keywords:** Type 1 diabetes, Interleukin-2, Disease register, Internet, Recruitment

## Abstract

**Background:**

A barrier to the successful development of new disease treatments is the timely recruitment of participants to experimental medicine studies that are primarily designed to investigate biological mechanisms rather than evaluate clinical efficacy. The aim of this study was to analyse the performance of three recruitment sources and the effect of publicity events during the A**d**aptive study of **IL**-2 dose on regulatory T cells in **t**ype **1 d**iabetes (DILT1D).

**Methods:**

The final study outcome, demography, disease duration, residence and the effect of publicity events on the performance of three recruitment sources (clinics, type 1 diabetes (T1D) disease register and the internet) were analysed from a bespoke DILT1D recruitment database. For the internet source, the origin of website hits in relation to publicity events was also evaluated.

**Results:**

A total of 735 potentially eligible participants were approached to identify the final 45 DILT1D participants. A total of 477 (64%) were identified via the disease register, but only 59 (12%) responded to contact. A total of 317 individuals registered with the DILT1D study team. Self-referral via the study website generated 170 (54%) registered individuals and was the most popular and successful source, with 88 (28%) sourced from diabetes clinics and 59 (19%) from the disease register. Of those with known T1D duration (N = 272), the internet and clinics sources identified a larger number (57, 21%) of newly diagnosed T1D (<100 days post-diagnosis) compared to the register (1, 0.4%). The internet extended the geographical reach of the study, enabling both national and international participation. Targeted website posts and promotional events from organisations supporting T1D research and treatment during the trial were essential to the success of the internet recruitment strategy.

**Conclusions:**

Analysis of the DILT1D study recruitment outcomes illustrates the utility of an active internet recruitment strategy, supported by patient groups and charities, funding agencies and sponsors, in successfully conducting an early phase study in T1D. This recruitment strategy should now be evaluated in late-stage trials to develop treatments for T1D and other diseases.

**Trial registration:**

NCT01827735 (registered: 4 April 2013); ISRCTN27852285 (registered: 23 March 2013); DRN767 (registered: 21 January 2013).

**Electronic supplementary material:**

The online version of this article (doi:10.1186/s13063-015-0583-7) contains supplementary material, which is available to authorized users.

## Background

The timely identification and enrolment of a pre-specified number of participants in a clinical trial is pivotal to the success or failure of a study. In the United Kingdom, 97% of the public believe the health service should support research into new treatments for disease, and 72% have expressed a preference that they would like to be offered the opportunity to participate in a clinical trial of a new medicine or treatment if they suffered from a condition that affected their day-to-day activities [1]. On the other hand, analysis of randomised controlled trials (RCTs) has found that approximately 50% of RCTs do not meet recruitment targets, and of those that complete recruitment only 50% do so on time, with little improvement in these outcomes in the last 30 years [2]. Furthermore, the most common reason for the discontinuation of RCTs involving patient participation is lack of recruitment, with up to 20% of studies prematurely stopping for this reason [3]. Yet there is limited published research to guide investigators on how to optimise recruitment and a paucity of data on how to successfully utilise the internet to improve patient participation in clinical trials [4,5].

Type 1 diabetes (T1D) is one of the most common chronic autoimmune diseases and is rapidly increasing in incidence [6]. Treatment of T1D requires life-long insulin therapy to limit microvascular complications (retinopathy, neuropathy and nephropathy), combined with intensive clinical monitoring and interventions to limit macrovascular complications (cardiovascular and cerebral vascular disease) [7]. Despite incremental improvements in care since the discovery of insulin therapy in 1922, current clinical outcomes remain suboptimal, with current intensive insulin therapies limited by hypoglycaemia and few patients able to achieve adequate long-term glycemic control to prevent end organ damage [8]. Insulin treatment, though life-saving, does not treat the underlying autoimmune pathogenesis leading to inevitable insulinopenia for most individuals and thereby a poorer prognosis [9]. Treatment with immunotherapy could potentially arrest the immune-mediated destruction of insulin-producing pancreatic beta cells to preserve endogenous insulin production, leading to lower exogenous insulin needs and increased resistance to hypoglycemia due to preservation of counter-regulatory endocrine pathways [10,11]. However, the promise of immunotherapy in T1D has not been realised in clinical practice to date, owing to an inability to translate successful treatments from rodent models of diabetes to T1D [12], the recurrent use of doses and treatment protocols in T1D of immunotherapies that were optimised for other indications [13], and an underestimation of the heterogeneity of both T1D and the response of participants to immunotherapy in clinical trials [14].

To address our current limited knowledge of the treatment of autoimmunity in T1D, we have initiated a mechanistic clinical study programme that combines novel study design with experimental medicine to understand how interleukin-2 (IL-2, aldesleukin, Proleukin®), a critical cytokine mediating immune activation and tolerance (unresponsiveness to self) in humans, modulates the immune system in T1D [15]. The rationale for investigating ultra-low dose (ULD) IL-2 treatment in T1D was provided by genome-wide association studies (GWAS) that have identified multiple genes in the IL-2-regulatory T (Treg) cell-T effector (Teff) cell pathways (*IL2RA* (CD25), *PTPN2*, *IL2*, *BACH2*, *CTLA4*, *IL21*, *IL6* and *IFIH1*) associated with the risk of development of T1D [16]. In addition, deep immunophenotyping has found that reduced expression of CD25 and reduced secretion of IL-2 by Teffs is associated with susceptibility to T1D [17]. This suggests that IL-2 replacement therapy could provide similar protection from disease progression as genetic resistance alleles, by enhancing IL-2-dependent regulatory Treg responses to restore dominant Treg suppression of autoreactive Teffs. By understanding the effects of ULD IL-2 in T1D patients *in vivo*, we can proceed with exploring its development as a potential immunotherapy for the disease in a more specific and mechanism-based approach [18].

The challenge of recruiting to mechanistic trials is that the intervention is designed to investigate a biological process and progress the development of a treatment that is more likely to benefit future patients when they are diagnosed with the condition, rather than the actual participants in the trial [19]. The statistical design of the DILT1D phase I study required a relatively large number of participants (N = 45) to be recruited to complete the study, compared to previous early phase studies of IL-2 therapy in T1D (N = 25) [20] and healthy individuals (N = 22) [21]. Therefore, three potential sources of participants were identified (the internet, clinics and register) during protocol development to maximise recruitment. Analyses of these sources have allowed comparison between how direct self-referral via the internet performed as compared to referral by health care professionals or from the T1D disease register. Overall, the recruitment for the DILT1D study exceeded expectations and the trial completed 11 months ahead of the planned 24-month duration. The analysis of patient participation in the DILT1D study indicates how to optimise recruitment of future studies in the programme and may assist others in designing recruitment strategies to mechanistic studies and late-stage trials that are needed to develop treatments for T1D and other diseases with unmet needs.

## Methods

### DILT1D study outline

The DILT1D study was a single centre, non-randomised, open label, mechanistic adaptive dose-finding study, consisting of 11 visits over nine weeks. In total, 45 participants with either newly (<100 days post-diagnosis) or recently diagnosed (< two years post-diagnosis) T1D were screened and 40 met the following inclusion criteria: T1D of less than two years duration, one autoantibody positive, aged 18 to 50 years and living in the European Union. The date of diagnosis of T1D was established by referring physicians, diabetes specialist nurses, review of register records and self-reporting by potential participants. Potential participants interested in enrolling in the study were provided with a patient information sheet and an informed consent form to review. Participants were given a minimum of 24 hours to consider the information provided and then were contacted to determine if they remained interested in participating in the study or if they had any further queries. Interested potential participants were then invited to attend for an appointment where the Chief Investigator (CI) or delegate discussed the study with the participant, who then provided informed consent.

Five participants were found to be ineligible at screening since they did not have a single T1D-associated autoantibody (anti-islet cell, anti-GAD, anti-IA2 or anti-ZnT8). Eligible participants were treated with a single dose of aldesleukin (Proleukin® – Novartis Pharmaceuticals, Camberley, UK) between 22 March 2013 and the early completion of the study owing to full recruitment on 15 May 2014 (planned duration was two years). Participants did not receive any payment for participating in the study, but did receive a reimbursed stipend for reasonable expenses incurred by their participation in the study (such as travel, parking, meals, accommodation and child care costs). DILT1D was designed as a single site study at the National Institute for Health Research (NIHR) Cambridge Biomedical Research Centre, since there was a requirement to carry out specialist time-dependant whole blood immunological assays within four hours of collection, in order to measure the primary and secondary outcomes. The rationale and study design have been published [15].

### The primary objectives of the DILT1D recruitment analysis

The aims of this recruitment analysis are to evaluate the performance of three pre-specified recruitment sources to optimise the recruitment strategy for the next study in the JDRF/Wellcome Trust Diabetes and Inflammation Laboratory (DIL) programme (DILfrequency [22]), and to share our methods with the broader clinical trials community.

### Recruitment sources

The three recruitment sources for the DILT1D study were:

1. Clinics: Potential participants were informed of the study by their physician, specialist diabetes nurse or research nurse at their diabetes clinic, either at Addenbrooke’s Hospital (Cambridge, United Kingdom) or at dedicated Participant Identification Centres (PICs) [23] in nearby hospitals. The contact details of potential participants were, with their agreement, forwarded to the DILT1D clinical study team (consisting of a physician, three research nurses and a data manager/developer).
2. Register: The After Diabetes Diagnosis Research Support System-2 (ADDRESS-2, UK Clinical Research Network identifier: UKCRN9689) [24] register enrols participants across England and Wales diagnosed with T1D in a disease-specific register designed to facilitate recruitment to clinical trials. The ADDRESS-2 protocol specified that the initial contact to the members of the ADDRESS-2 register was made by local research nurses. The personal contact details of interested potential participants were then passed to the DILT1D team by ADDRESS-2 staff. Also included in this source were potentially eligible participants from the completed observational study Diabetes-Genes, Autoimmunity and Prevention (D-GAP, UK Clinical Research Network identifier: UKCRN5798; Diabetes Research Network identifier: DRN 231) [25] that had consented to be contacted about future T1D research studies. These individuals were contacted by a D-GAP staff member and, if interested in DILT1D, their details were passed to the DILT1D team.
3. Internet: The internet was identified as a potential source of direct recruitment by the DILT1D team. The website (http://www.clinical-trials-type1-diabetes.com/) was developed, along with an associated Facebook page (https://www.facebook.com/ClinicalTrialsType1Diabetes) and Twitter feed (https://twitter.com/t1diabetestrial), both of which were updated on a fortnightly to monthly basis to provide content and public engagement during the study. Potential participants provided their contact details directly via the website to the study team. Details of the study website and social media were promoted by placing articles on diabetes charities websites [26-28], a joint press release from the study funders and the University of Cambridge [29], by public talks to T1D patients and the placement of the DILT1D website address, Quick Response (QR) Code for the URL and contact details on the publicity leaflets and posters placed at hospital, talks and clinics.

These potential recruitment sources had been identified prior to initiation of the study and were included in the study protocol.

### Management and strategy

A systematic approach was developed to manage and monitor recruitment to the study. Individual level recruitment data collection for potential study participants was facilitated by a locally developed recruitment and contact management database application. The application had four essential requirements:

1. The ability to securely store and record demographic (name, address and so forth) and eligibility data (such as duration and diagnosis status) on registered individuals.
2. To provide an audit of individuals’ status change over time.
3. To provide a contact management facility that included contact between the trial team and participants and within the trial team.
4. To provide a method by which participant schedules could be:
  4.a generated automatically,
  4.b shared with potential participants individually and
  4.c publicised more widely with the trial team by writing to other shared calendar systems.

The application is available on request for comparative purposes.

The functionality of this application was updated throughout the course of the study to facilitate data flows within the research unit and maximise time that the team spent on recruitment activities, particularly in pre-defining participant visit schedules and optimising study team correspondence to participants. Individual research nurses were allocated responsibility to optimise recruitment from each of the three sources. A weekly recruitment meeting was held and chaired by the CI to coordinate and facilitate recruitment to the study.

### Recruitment data

The database was explored for identifying relationships between the recruitment source, the study outcomes, demography, date of diagnosis and time to recruitment of potential participants. Each potential participant was defined as belonging to one of five ‘outcome’ categories, depending on their status after the recruitment process had ended.

The five study recruitment outcomes were:

1. Consented: The individual consented to participation in the study.
2. Declined: The individual declined to participate in the study.
3. Ineligible: The individual was outside the inclusion criteria.
4. Not permitted: Eligible individuals who lived outside of the European Union and others who were not able to enrol as the study had been completed.
5. Non-communicator: Registered individuals who were lost to contact.

### Socioeconomic analysis

Socioeconomic data (such as employment, education and income status) on individual potential participants was not available since this was not part of the eligibility criteria of the study. Instead, area level socioeconomic indicators (SEI) were applied to potential participants where an English postcode was available [30,31]. As such, data should be interpreted as ‘participants coming from areas of a particular deprivation status’. The reliance on full postcode data meant that the Index of Multiple Deprivation (IMD) was available for 47% of potential participants (149 out of 317). The majority of missing data was due to the absence of such information from internet-sourced participants (28 out of 170, 16%), whilst clinics (70 out of 88, 80%) and register (51 out of 59, 86%) groups were more complete.

### Website analysis

Aggregate data on the use of the study website and records of where each visit to the study website originated from, the length of time spent at the site and ‘direct’ visits (placement of the URL directly into their browser or use of the QR by mobile device or bookmarking the site) was sourced from Google Analytics [32]. For comparison, the total number of referral sites was grouped into similar categories (Additional file 1). For example, referrals from Facebook and Twitter were given a category, whereas other sites of similar content or domain names were grouped (for example, ‘_’.cam.ac.uk, grouped as University of Cambridge).

### Statistical analyses

Age comparisons between gender and recruitment source groups used non-parametric methods (Mann-Whitney U and Kruskal-Wallis tests) on account of the non-normal distribution of age in this cohort (D’Agostino omnibus K^2^ = 8.245, *P* = 0.0162). A relatively small group (N = 19) of minors (<18 years) identified by the internet source only were excluded from this analysis since the referral was made by their parents or guardians. ‘Goodness-of-fit’ and independence tests were performed to compare proportions of respondents within recruitment source. The ‘not permitted’ group of participants (N = 20) were excluded from tests of independence of trial outcome and recruitment source as expected frequencies were less than five [33]. To compare the impact of individual events, we followed a previously used approach [5] where weekly registration rates were compared before and after publicity events. For the first four events, weekly registration rates were measured between each event over a one-month period. For the period after the fourth and before and after the fifth events, weekly rates over a five-week period were considered. The average weekly registration rate and the range of weekly registration rates over the estimated period are reported.

### Ethical approval and sponsorship

The trial was sponsored by the University of Cambridge and Cambridge Universities Hospital NHS Foundation Trust. Ethical approval for the study was granted by the Health Research Authority, National Research Ethics Service, England (approval number: 13/EE/0020) on 18 February 2013.

## Results

### Demography and type 1 diabetes duration of potential trial participants

**Figure 1:**
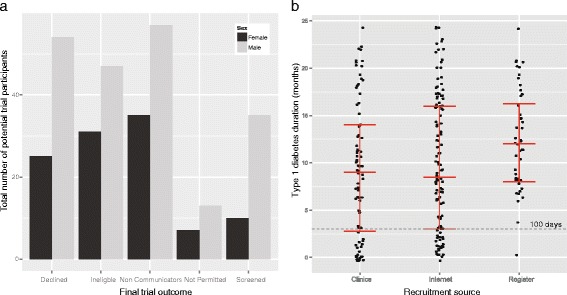
**Gender and duration of type 1 diabetes of eligible potential participants in DILT1D. a)** Males and females were equally likely to decline to participate as to enrol, with similar proportions observed across the final trial outcomes regardless of recruitment source. **b)** Both the clinics and internet sources were effective in identifying newly diagnosed cases of T1D (less than 100-day duration) compared to the register. A total of 19 individuals were registered at diagnosis (clinics = 12, internet = 6, register = 1). The internet source identified the largest number of cases but the clinics source was the most efficient method of locating eligible participants. Median and interquartile range shown for 246 eligible participants with less than two years duration of type 1 diabetes.

We observed a ratio of 1.9:1 male-to-female potential participants, in agreement with the previously reported skewing towards male T1D patients versus female ones [34]. Similar numbers of male and female potential participants (data available for 314 participants) were observed for each recruitment source (${\chi}_2^2$ = 1.9903, N = 314, *P* = 0.3697) (Table 1), as well as for the final trial outcome of participants (${\chi}_4^2$ = 4.7545, N = 314, *P* = 0.3134) (Figure 1a).

**Table 1:** **Baseline demographics of registered potential participants for DILT1D stratified by recruitment source**

**Baseline demographics**	**Clinics**	**Internet**	**Register**
Age	N = 88; N = 84^a^	N = 170; N = 121^a^	N = 59; N = 48^a^
	30 (24 - 37.25)^b^	26 (19 - 35)^b^	31 (23.75 - 35)^b^
Gender (F/M)	N = 88	N = 170; N = 168^a^	N = 59; N = 58^a^
	29/59	63/105	16/42
Distance from trial site (km)	N = 88; N = 76^a^	N = 170; N = 72^a^	N = 59; N = 54^a^
	23.62 (9.302 - 51.450)^b^	150.30 (71.89 - 478.2)^b^	123.60 (69.21 - 202.50)^b^
Index of Multiple Deprivation (IMD) score	N = 88; N = 70^a^	N = 170; N = 28^a^	N = 59; N = 51^a^
	9.19 (6.448 - 16.17)^b^	13.38 (8.548 - 24.75) ^b^	15.84 (8.235 - 29.88)^b^

^a^Number of individuals where data is available.

^b^Median (interquartile range).

Similar ages (data available for 253 participants) were observed for male and female potential participants (U = 6336.5, N = 253, *P* = 0.1886) and for the potential participants identified by each recruitment source (Kruskal-Wallis *χ*^2^ = 5.1844, N = 253, *P* = 0.0749). Potential participants who were identified by the register and internet sources lived further from the trial site (data available for 202 participants) (Kruskal-Wallis *χ*^2^ = 93.5475, N = 202, *P* <0.0001). Those identified from the clinics source (data available for 149 participants) came from less deprived areas (Kruskal-Wallis *χ*^2^ = 10.1629, N = 149, *P* = 0.0062), having a lower deprivation score compared to the other sources, though this analysis is limited by the lack of postcode data available for the internet source (Table 1).

Generally, trials of immunotherapies to treat newly diagnosed T1D state in their eligibility criteria that participants are recruited within 100 days of diagnosis. Though this group was not specifically targeted for recruitment to DILT1D, both the internet and clinics sources identified a relatively large number of newly diagnosed cases. Out of the 272 participants with a known duration of T1D, 57 (21%) newly diagnosed T1D cases were identified from these sources, a number that corresponds to 23% of the eligible participants (a total of 246 participants with less than two years disease duration since diagnosis). Only one (0.4%, N = 246) newly diagnosed T1D participant was identified by the register (Figure 1b, Table 2). Overall, when all potential participants (irrespective of whether duration was known) are included, the clinics sources (observed versus expected: 24 versus 16) outperform the internet (33 versus 31) and register (one versus 11) (${\chi}_2^2$ =15.762, N = 317, *P* = 0.0004).

**Table 2:** **Duration of disease by source at registration with DILT1D study team**

**T1D duration**	**Clinics**	**Internet**	**Register**
**Less than 100 days** ^**a**^	24 (9%)	33 (12%)	1 (0.4%)
**Between 100 days and two years** ^**b**^	56 (21%)	85 (31%)	47 (17%)
**Two years or more**	1 (0.4%)	24 (9%)	1 (0.4%)

^a^Newly diagnosed T1D.

^b^Recently diagnosed T1D.

Numbers given are of individuals in each group category where data was available (N = 272).

### Analysis of trial outcome of participants by recruitment source

A total of 735 potential participants were directly contacted by, or approached on behalf of, the study team regarding the DILT1D trial during the 13-month study period.

Outcome of contacts by register: The majority of the potential eligible participants were identified by the use of the ADDRESS-2 register (477 out of 735, 64%), but a small proportion of them responded to the contact (59 out 477, 12%) and commenced direct communication with the DILT1D study specific team. In the case of the D-GAP study, 14 eligible individuals were identified and contacted, with a high proportion responding to the contact (12 out of 14, 85%) and then proceeding to register with the DILT1D team (these 12 are included in the 59 ‘responded to contact’ participants).

**Figure 2:**
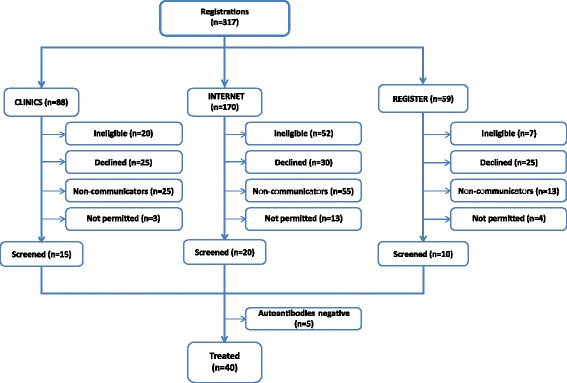
**Consort diagram for potential DILT1D study participants registered on the DILT1D database until the point of treatment.** The most successful method of registering potential participants was via self-referral from the study website (internet). Once individuals registered with the study team, similar proportions from each source proceeded to trial enrolment. Participants who were negative for autoantibodies were from the clinics (N = 2), internet (N = 2) and register (N = 1) (not permitted - outside the EU or registered after last participant treated, non-communicators - initially registered but did not respond to messages from DILT1D team).

Outcome of contact by the DILT1D study team: From the three recruitment sources, a total of 317 individuals initially communicated with the DILT1D team and were registered on the study database (Figure 2). The final trial recruitment outcome was influenced by the source of potential participants (${\chi}_6^2$ = 20.6702, N = 297, *P* = 0.0021). The most successful method of gaining interest in DILT1D and providing immediate direct contact with the study team was via self-referral by the internet (170 out of 317, 54%), although the largest proportion of ineligible and not-permitted participants (Additional file 2) was also observed via this source (65 out of 170, 38%) compared with clinics (23 out of 88, 26%) and the register (11 out of 59, 19%). The primary reasons for ineligibility from the internet source were age and duration of T1D outside the inclusion criteria (Table 3).

**Table 3:** **Reason for ineligibility by recruitment source**

**Source**	**Age**	**Thyroid disorder**	**Diagnosis**	**Duration** ^**a**^	**Drug**	**Malignancy <5 yrs**	**Medical history**	**Pregnancy/Breast feeding**
Clinics	2 (2.3%)	3 (3.4%)	2 (2.3%)	11 (13%)	0 (0%)	0 (0%)	0 (0%)	2 (2.3%)
Register	1 (1.7%)	2 (3.4%)	1 (1.7%)	2 (3.4%)	1 (1.7%)	0 (0%)	0 (0%)	0 (0%)
Internet	25 (15%)	0 (0%)	0 (0%)	24 (15%)	0 (0%)	1 (0.6%)	1 (0.6%)	1 (0.6%)

^a^11 participants who registered with the DILT1D study team had a duration of T1D of between 20 and 24 months. This group were unable to enrol in the trial before they became ineligible.

Proportion of individuals declining enrolment by recruitment source: The largest proportion of potentially eligible participants that declined enrolment after communicating with the study team was observed from the register (25 out of 59, 42%), compared to the clinics (25 out of 88, 28%) and the internet (30 out of 170, 18%). The main reason for declining enrolment across all sources was lack of time or concurrent personal commitments (Table 4).

**Table 4:** **Reason for declining study participation**

**Source**	**Time commitments**	**Location**	**Not interested**	**Other**	**Drug concerns**
Clinics	13 (16%)	1 (1%)	8 (10%)	2 (3%)	1 (1%)
Register	15 (19%)	6 (8%)	1 (1%)	3 (4%)	0 (0%)
Internet	18 (23%)	7 (9%)	3 (4%)	2 (3%)	0 (0%)

Analysis of success of each recruitment source: The success of recruitment from each source was assessed by calculating the ratio of screened individuals over the total number of trial potential participants within each source that had registered with the trial team. The proportions of success of the three sources appeared to be similar (${\chi}_2^2$ = 1.7788, N = 317, *P* = 0.4109), with the success of the register (10 out of 59, 16%) and clinics (15 out of 88, 17%) being higher than the corresponding one of the internet (20 out of 170, 12%). However, if the total number of individuals contacted by the register (N = 477) is included for calculating the success rate of the register out of the total number of identified potentially eligible participants, these proportions appear to differ ${\chi}_2^2$ = 41.1354, N = 735, *P* <0.0001), with the proportion of success from the register to be the smallest (10 out of 477, 6%).

### The internet as a recruitment tool to extend the geographical reach of DILT1D

**Figure 3:**
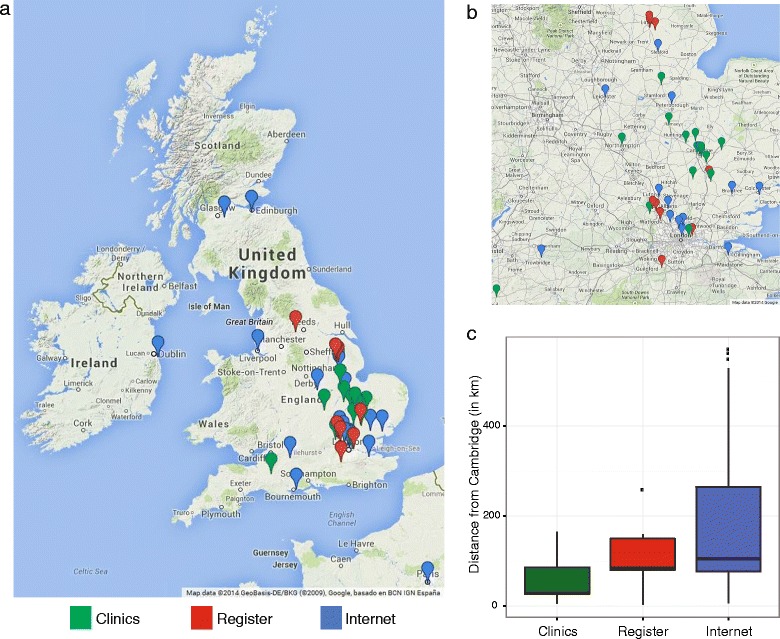
**Geographical spread of DILT1D study participants residence by recruitment source (N = 45). a)** The internet source (blue) extended the geographical reach of the study, allowing international participation. **b)** The clinics (green) and register sources (red) enabled local and national recruitment. **c)** On average, participants recruited from the internet source (204.1 km) travelled further on average than those recruited from the clinics (54.7 km) or register (105.9 km).

The internet-based recruitment source led to the enrolment of participants in the study from outside the immediate catchment area of the NIHR Cambridge Biomedical Research Centre, the PIC sites and the ADDRESS-2 register, which is confined to England and Wales (Figure 3). Four individuals self-referred via the internet from Scotland, Ireland and France and travelled to Cambridge to participate in the study. Participants recruited from the internet (expenses expressed as median (interquartile range), £767.4 (439.4 to 1413)) and register (£787.7 (298.2 to 1042)) claimed more expenses than participants recruited from the local clinics (£268.6 (133.6 to 346.2)). Analysis of the enrolment from the three recruitment sources found that the internet-recruited participants lived on average 204.1 km (total range: 5.60 to 570 km) from Cambridge, further than those recruited from the clinics (54.7 km) and register (105.9 km) (F = 5.3899, N = 45, *P* = 0.0082), respectively. There were three missed visits for the entire study, two from clinics sources (from participants living 20 km and 7 km from Cambridge), and one from the internet source (from a participant living 62 km from Cambridge). This suggests that the internet recruitment approach was successful in extending the geographical area for enrolment of participants to the study, since these participants travelled further compared to more traditional methods of local enrolment to a single site study.

### Analysis of the origin of website hits

**Figure 4:**
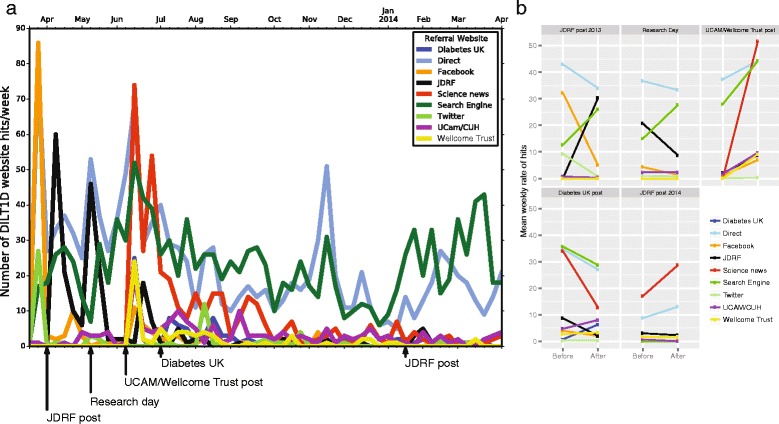
**Analysis of website traffic for the DILT1D study website. a)** Website activity showing the number and origin of website hits per week and the relationship to publicity events undertaken during the trial. **b)** The average number of weekly website referrals before each publicity event and after, showing that the joint press release from the University of Cambridge and The Wellcome Trust had the greatest impact on website activity, with much of that increase being from science news at Wired.

To inform potential participants of the DILT1D trial we engaged over the course of the study with Diabetes UK, the Juvenile Diabetes Research Foundation (JDRF), the Wellcome Trust and the University of Cambridge, as well as presenting at the Addenbrooke’s Hospital’s annual T1D study day (11 May 2013). By analysing activity on the website clinical-trials-type-1-diabetes.com, it was possible to determine the origin of website hits (referrals) and the effect of publicity (Figure 4). The posting of information regarding the trial with the web address on the Diabetes UK and JDRF websites increased the referral rate from these sites, though the second post by the JDRF that gave an update on the progress of the trial had less impact (Figure 4). The joint press release post from the Wellcome Trust and the University of Cambridge had the greatest impact on website activity, with much of that increase stemming from search engines, science news at Wired [35], as well as the Diabetes UK, the Wellcome Trust and the University of Cambridge websites.

### The role of publicity events in increasing DILT1D database registrations

**Figure 5:**
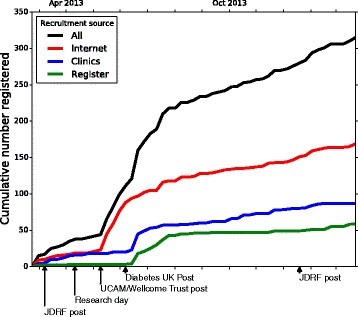
**Cumulative registration of potential DILT1D participants.** Individual publicity events were observed to impact different recruitment sources with the University of Cambridge and Wellcome Trust increasing internet registration, while the Diabetes UK post increased the clinics and register sources.

Most of the potential study participants (175 out of 317, 55%) came to the attention of the study team in a three-month period between June and August 2013. After an initial surge in the first week of the study, the weekly registration rate remained relatively low, but then increased following the JDRF post (Figure 5). The Wellcome Trust and the University of Cambridge joint press release increased database registration six to seven fold (1.5 (0-2) versus 16.8 (12-22), expressed as average weekly rates with their associated ranges over the estimated periods given in the brackets). Similarly, after the Diabetes UK post there was a surge of registrations in the first week after this event, with the highest weekly registration rate for the entire study (39 registrations). The final JDRF post had little impact on registration, with the level having dropped back to that of after the first week of the study (Figure 5). Analysis of registration rate via recruitment source suggested that the Wellcome Trust and the University of Cambridge joint press release was most effective in increasing number of registrations from the internet (1 (0-2) versus 16.2 (12-22)), whilst the Diabetes UK post increased the number of registrations from the clinics (1.4 (0-5) versus 6.8 (1-22)) and register sources (0 (0-0) versus 5.4 (1-14)) (average weekly rates with their associated ranges over the estimated periods given in the brackets).

## Discussion

The DILT1D study successfully achieved enrolment of the study in a timely and efficient manner, with the study completing 11 months earlier than the two-year protocol specified target, with a recruitment rate of 3.3 participants per month that exceeded previous studies of IL-2 therapy in T1D (2.3 per month) [20] and healthy individuals (one per month) [21]. Nevertheless, there is limited published research on effective recruitment strategies to intensive mechanistic studies or trials, despite the importance of these studies in understanding human physiology, pathophysiology and drug responses. The DILT1D (single dose) study was the first study of a program to understand the mechanism of action of ULD IL-2 in participants with T1D. The study has now been followed by the ongoing DILfrequency study (repeat dose) that aims to establish the optimal dosing interval for ULD IL-2 therapy [22]. The results from these studies will inform the treatment protocols for future phase II and III RCTs of ULD IL-2 therapy, thereby maximising the chance of success in these trials based on a prior understanding of the effects of this immunotherapy on the human immune system. Key to this strategy is the rapid conduct of the early phase studies by optimising enrolment. To improve enrolment to current and future studies in T1D, we have analysed the DILT1D study recruitment data from the clinics, register and internet sources and evaluated the effectiveness of each method.

The T1D register ADDRESS-2 identified the most potential participants for the DILT1D study, but only a small proportion of these individuals responded to contact by the register staff and commenced direct communication with the study team. The largest proportion of eligible participants declining enrolment after communication with the study team also came from the register, with potential participants identified from the register more likely to decline participation than from the clinics or internet sources. The reasons for declining study participation were similar across sources, with time commitment being the principal reason. Overall, the data showed the T1D register, given the total number of potential participants contacted by both the register and DILT1D teams, to be the least effective source to enrol participants to the trial. The relative low yield of the register source for recruitment to DILT1D was unexpected since this group had already expressed an interest in participation in T1D research. One possible explanation is that the initial contact was not being made by the DILT1D study specific team.

The DILT1D study found that self-referral via the study website was the most popular and most successful recruitment source by which individuals came to the attention of the study team. More ineligible individuals were found through the internet source than the clinics and registry sources, but this was not unexpected since both the register and clinics sources had pre-screened potential participants to establish if they fulfilled the inclusion criteria. Slightly more females came to the attention of the study team after having discovered the study online, but on analysis similar proportions of males and females were observed between the three recruitment sources. It might have been expected that internet-sourced respondents would be younger, however, the online source also provided the oldest people to be registered on the database, and the age range of respondents was similar to the other sources. Importantly, once an individual from any source had been registered on the DILT1D recruitment database, they were equally likely to proceed to enrolment, indicating the importance of the study specific team in communicating directly with potential participants.

The clinics source was found to be best at identifying newly diagnosed T1D compared to the internet and register sources. However, when ineligible individuals are excluded from all recruitment sources, both the clinics and internet sources identified more newly diagnosed than the register. This suggests that the internet may be good method of identifying newly diagnosed participants by self-referral for experimental medicine studies and immunotherapy intervention trials in T1D, but the study team will be required to screen out a high number of ineligible participants. The register was the least successful in identify this group, however this may be related to a time lag that may occur between registration on the register, and further contact and enrolment in the actual study. It may be possible to optimise recruitment of newly diagnosed participants from the register by allowing study teams to directly contact potential participants that have joined the register.

Multicentre international clinical trials potentially increase recruitment to a study by making the study available to a greater participant population by reducing the length of travel to a treatment site [36]. They do, however, increase the complexity of the trial management and logistics, and if a limited number of participants are recruited by each site, this reduces the ability for physicians to make clinical observations [37]. The DILT1D study was designed as a single site study to simplify trial management, to reduce variability in real-time whole blood immunological assays and to maximise clinical observations. A risk was that the trial would not recruit to schedule, but we found that use of a single trial site did not impede enrolment, and each recruitment source identified participants from different geographical areas. The clinics identified local participants, the register identified individuals in the region and the internet further increased the geographical reach of the study, allowing participants to self-refer nationally and internationally to the study.

During the DILT1D study the team undertook publicity events by partnering with funders, patient charities and the sponsor to actively promote the study. By analysis of the study website and the local database, the impact of these events on identifying potential participants to the study team was explored. Whilst all events increased the internet activity, it was only after certain events that this translated into increased registration on the study database. Individual posts from the JDRF were successful in increasing trial website activity and initially led to increases in internet registration rates that declined following the first event. A local research day presentation, though increasing search engine internet activity, had little effect on registrations, whilst the Wellcome Trust and University of Cambridge press release, which was featured on Wired, had a dramatic effect on referrals from other websites and led to a surge in the number of registrations from the internet source. On the other hand, the Diabetes UK post increased registrations from clinics and registry sources, possibly having stimulated interest from clinicians and research nurses, indicating that targeted online recruitment efforts can enhance recruitment from traditional sources. These findings suggest that an active recruitment strategy focused on the candidate study populations via press and/or post releases on disease-specific, charity, university and science, medicine and technology websites is effective in informing and enrolling participants in mechanistic studies.

Despite widespread use and access to the internet in developed countries, there is limited published data to guide investigations on how to successfully employ this media to optimise participation in clinical trials. Our development of an effective internet-based method to inform and engage potential participants in the DILT1D study suggests that this strategy could be generalizable to RCTs and late-stage trials to develop treatments for T1D and other diseases. In the future, we would encourage other investigators to utilise this active internet recruitment strategy prospectively to determine if it enhances participation, and thereby accelerates trial conduct.

## Conclusions

Management of recruitment to clinical trials is an essential component that leads to the successful conduct of studies to refine or develop new treatments for human disease. Analysis of our recruitment and internet data from the DILT1D study has found that multiple recruitment sources were needed, supported by an active internet recruitment strategy that required input from patient groups and charities, funders and the sponsors to engage participants during the study. We found that most potential participants utilised the internet for making contact with the study team compared to traditional recruitment modalities, and that this group did not find distance or borders a barrier to participation.

### Ethical approval, trial registration and commencement of enrolment

Ethical approval for the study was granted on 18 February 2013 by the Health Research Authority, National Regulatory Ethics Service (approval number: 13/EE/0020). The trial was approved for inclusion in the clinical research network portfolio (Diabetes Research Network 767) on 21 January 2013. The trial was registered with International Standard Randomised Controlled Trial Number Register (identifier: ISRCTN27852285) on 26 March 2013 and at Clinicaltrials.gov (identifier: NCT01827735) on 4 April 2013. The first participant was recruited on 8 March 2013, while the final participant completed the trial on 15 May 2014.

## Additional files

Additional file 1:
**DILT1D website referral groupings.** Description: List of all study website referral sources and associated grouping category. Additional file 2: Table S1. Not permitted by recruitment source. Description: Summary of criteria for assignment to non-permitted outcome group.

## Abbreviations

ADDRESS-2: The after diabetes diagnosis research support system-2
CI: Chief investigator
D-GAP: Diabetes - genes, autoimmunity and prevention
DIL: JDRF/Wellcome Trust Diabetes and Inflammation Laboratory
DILT1D: Adaptive study of IL-2 dose on regulatory T cells in type 1 diabetes
DRN: Diabetes Research Network
GWAS: Genome wide association study
IL-2: Interleukin 2
IMD: Index of multiple deprivation
JDRF: Juvenile diabetes research foundation
NCT: National Clinical Trials (number)
NIHR: National Institute for Health Research
PIC: Participant identification centre
QR: Quick response
RCT: Randomised controlled trial
SEI: Socioeconomic indicators
T1D: Type 1 diabetes
Teff: Effector T cell
Treg: Regulatory T cell
UKCRN: UK Clinical Research Network
ULD: Ultra-low dose
